# Direct observation of prion protein oligomer formation reveals an aggregation mechanism with multiple conformationally distinct species†
†Electronic supplementary information (ESI) available: Additional methods and materials, supplementary figures. Representative TIRF images of PrP aggregates at different times. (Fig. S1), single-aggregate TIRF measurements of PrP aggregates during fibril formation in the presence of 2 M GdnHCl (Fig. S2). See DOI: 10.1039/c8sc05627g


**DOI:** 10.1039/c8sc05627g

**Published:** 2019-03-25

**Authors:** Jason C. Sang, Ji-Eun Lee, Alexander J. Dear, Suman De, Georg Meisl, Alana M. Thackray, Raymond Bujdoso, Tuomas P. J. Knowles, David Klenerman

**Affiliations:** a Department of Chemistry , University of Cambridge , Lensfield Road , Cambridge , CB2 1EW , UK . Email: dk10012@cam.ac.uk; b Department of Veterinary Medicine , University of Cambridge , Madingley Road , Cambridge , CB3 0ES , UK

## Abstract

The aggregation of the prion protein (PrP) plays a key role in the development of prion diseases. In the past decade, a similar process has been associated with other proteins, such as Aβ, tau, and α-synuclein, which participate in other neurodegenerative diseases. It is increasingly recognized that the small oligomeric species of aggregates can play an important role in the development of prion diseases. However, determining the nature of the oligomers formed during the aggregation process has been experimentally difficult due to the lack of suitable methods capable of the detection and characterization of the low level of oligomers that may form. To address this problem, we have utilized single-aggregate methods to study the early events associated with aggregation of recombinant murine PrP *in vitro* to approach the bona fide process *in vivo*. PrP aggregation resulted in the formation of thioflavin T (ThT)-inactive and ThT-active species of oligomers. The ThT-active oligomers undergo conversion from a Proteinase K (PK)-sensitive to PK-resistant conformer, from which mature fibrils can eventually emerge. Overall, our results show that single-aggregate methods can provide structural and mechanistic insights into PrP aggregation, identify the potential species that mediates cytotoxicity, and reveal that a range of distinct oligomeric species with different properties is formed during prion protein aggregation.

## Abstract 2

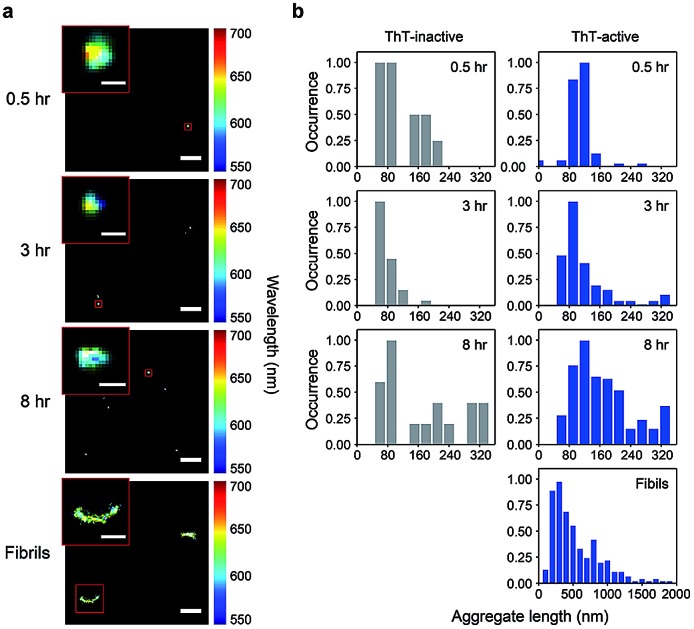
The aggregation of the prion protein (PrP) plays a key role in the development of prion diseases.

## Introduction

Prion diseases, such as Creutzfeldt–Jakob diseases of humans, bovine spongiform encephalopathy of cattle, and scrapie of sheep, are a class of lethal neurodegenerative diseases. These conditions are characterized by the accumulation of PrP^Sc^, an abnormal aggregated conformer of the normal host protein PrP^C^.^1^ Prion diseases are transmissible between individuals of the same or different species. The ‘protein-only’ hypothesis states that the transmissible prion agent comprises solely of PrP^Sc^.^2^ The structural conversion of PrP involves the formation of the fibrillar state of aggregates, which is generally considered to be relatively resistant to Proteinase K (PK) and contains a high cross-β sheet architecture.^3^–^5^ Despite numerous studies in the last decades, the molecular events involved in the aggregation process remain poorly defined. Increasing evidence argues that other disease-associated proteins, such as Aβ, tau, and α-synuclein, also share a similar aggregation mechanism with PrP^6^–^11^ that is classified as a ‘prion-like’ mechanism.

The early stage of fibril formation has been associated with low-molecular weight intermediates known as oligomers, which is likely to be structurally heterogeneous and highly toxic to cells.^12^,^13^ From biophysical studies in α-synuclein and yeast prions, the oligomeric species has been revealed to undergo a transition to a structurally more organized conformation and is able to grow into fibrillar species.^13^–^15^ In mammalian prion research, it remains unclear if this process occurs. Previous studies have characterized recombinant PrP oligomers with various approaches *in vitro* and shown that these are kinetically stable and off the pathway to form fibrils.^16^–^21^ However, depending on the conditions, the generation of oligomeric species of PrP could result in various conformations and kinetic properties. It is increasingly recognized that multiple conformers of PrP^Sc^ may exist, including PK-sensitive and PK-resistant species.^22^–^26^ Small soluble oligomeric species of PrP^Sc^ have been shown to be the most efficient mediators of prion infectivity^27^ and exert higher cytotoxicity than mature fibrils both *in vitro* and *in vivo*.^28^

The characterization of early events associated with the aggregation process is extremely challenging, since these aggregated species are highly heterogeneous and exist in a transient manner. A recent study using the yeast prion Ure2 has shown that a single-aggregate analysis can provide a new and informative approach to this complex area of prion biology and establish a temporal relationship between the oligomeric and mature fibrillar species.^29^ Two structurally distinct oligomeric species of Ure2 were identified that occur before fibril formation. In the case of mammalian PrP, prion infectivity and neurotoxicity were suggested to involve different protein aggregate species that appeared with different kinetics,^22^,^30^ while the species that contribute to the production of neurotoxicity were still undefined. The proportional contribution of PK-sensitive and PK-resistant PrP^Sc^ to these oligomers remains unclear. Furthermore, there is little knowledge of the structural heterogeneity and physical properties of PrP oligomers that form at the early stage of PrP aggregation.

Aggregation-prone proteins in various neurodegenerative diseases share a similar molecular phenomenon of nucleation, growing, templating, and spreading. It is fundamentally important to establish the nature and kinetics of misfolded protein aggregation. The understanding of the molecular details of the aggregation process and the identification of toxic species of aggregates can contribute to potential therapeutic targets to halt or retard their accumulation and resultant toxicity. In our studies reported here, we have investigated the structural transition of the oligomeric species formed during aggregation of full-length recombinant murine PrP using single-aggregate approaches. The application of this novel approach has provided new insights into the early stages of PrP aggregation *in vitro*, identifying five oligomeric species with distinct structural properties. With the use of the kinetic modeling to the data, we have developed a multi-step kinetic scheme for the early stage of fibril formation of recombinant PrP and described the time evolution of the oligomers observed in a quantitative manner. These findings illustrate the complexity of PrP aggregation *in vitro* and provide a possible aggregation mechanism for further studies *in vivo*.

## Results

### Single-aggregate imaging reveals the gradual formation of small aggregates in early PrP aggregation

Mouse PrP aggregation was performed at 37 °C with 200 rpm under partially denaturing conditions of 2 M GdnHCl. Using single-aggregate imaging based on total internal reflection fluorescence microscopy (TIRFM),^31^ the aggregation reaction was followed by taking aliquots at different time points from the reaction mixture and mixing with 25 μM thioflavin T (ThT) for imaging (Fig. S1†). The solubility of full-length PrP restricted the range of monomer concentrations applied. From 22.5 to 32.5 μM of the monomer concentration, we observed that the overall intensity of PrP aggregates formed at the early stage of aggregation (*t* < 8 h), as well as their total number, gradually increased (Fig. S2a and b†). We also found that the rate of increase of aggregate number depends on the initial protein concentration. Only aggregates smaller than the diffraction limit of 450 nm were detected at early stages of aggregation, and no fibrils were detected until 24 h of aggregation. This result is consistent with previously measured kinetic data using a bulk solution under the same conditions.^32^

### Early-formed PrP oligomers are structurally diverse

We have previously shown that spectrally-resolved PAINT (Points Accumulation for Imaging in Nanoscale Topography), or sPAINT, enables super-resolution imaging of protein aggregates with a spatial resolution of 40 nm, as well as probing their surface hydrophobicity.^33^ The spectral shift of the polarity-sensitive fluorescent dye Nile red (NR), which transiently and non-specifically binds to protein aggregates, allows the measurement of relative surface hydrophobicity of individual protein aggregates. The blue-shift of the NR emission indicates a more hydrophobic surface, while the red-shift indicates a more hydrophilic surface. sPAINT can be combined with ThT imaging,^34^ making it possible to characterize PrP aggregates based both on their amyloid structure and their hydrophobicity. It is also possible to distinguish ThT-inactive species from ThT-active species. To gain more insights into the early stages of PrP aggregation, we applied this approach to visualize the morphology of PrP aggregates, as well as to characterize the temporal change in surface hydrophobicity. Aliquots at different time points were taken from an aggregation reaction, diluted to 0.1 μM, and then loaded onto a coverslip for sPAINT experiments.

**Fig. 1:**
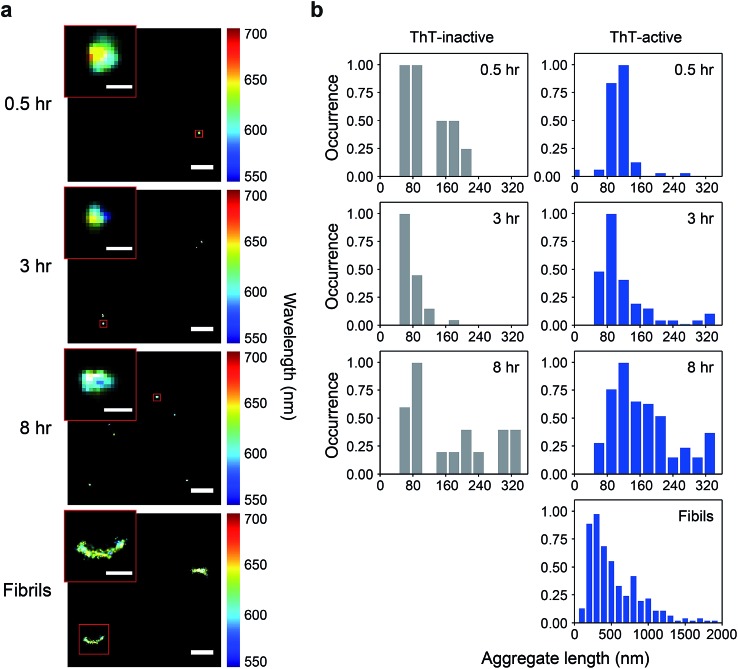
(a) Super-resolved sPAINT images of PrP aggregates with Nile red (NR) at different time points. Monomeric PrP was incubated in a 1.5 mL Eppendorf tube with 2 M GdnHCl at 37 °C with 200 rpm. At various time points, aliquots were removed from the reaction mix and adsorbed onto a glass coverslip for sPAINT imaging. The images are colored by the wavelength of individual NR fluorescence signals. The scale bars represent 1 μm, and those in the insets are 100 nm for 0.5, 3, and 8 h and 500 nm for fibrils at 48 h. (b) Length distribution of ThT-inactive and ThT-active PrP species at different time points. The distributions shown correspond to the combined results of three independent measurements. The maximum value in each distribution was normalized to 1. The overall number of analyzed PrP aggregates is as follows. At 0.5 h: *N*_ThT-inactive_ = 13, *N*_ThT-active_ = 67; at 3 h: *N*_ThT-inactive_ = 33, *N*_ThT-active_ = 165; at 8 h: *N*_ThT-inactive_ = 17, *N*_ThT-active_ = 219; for fibrils at 48 h: *N*_ThT-active_ = 231.

**Fig. 2:**
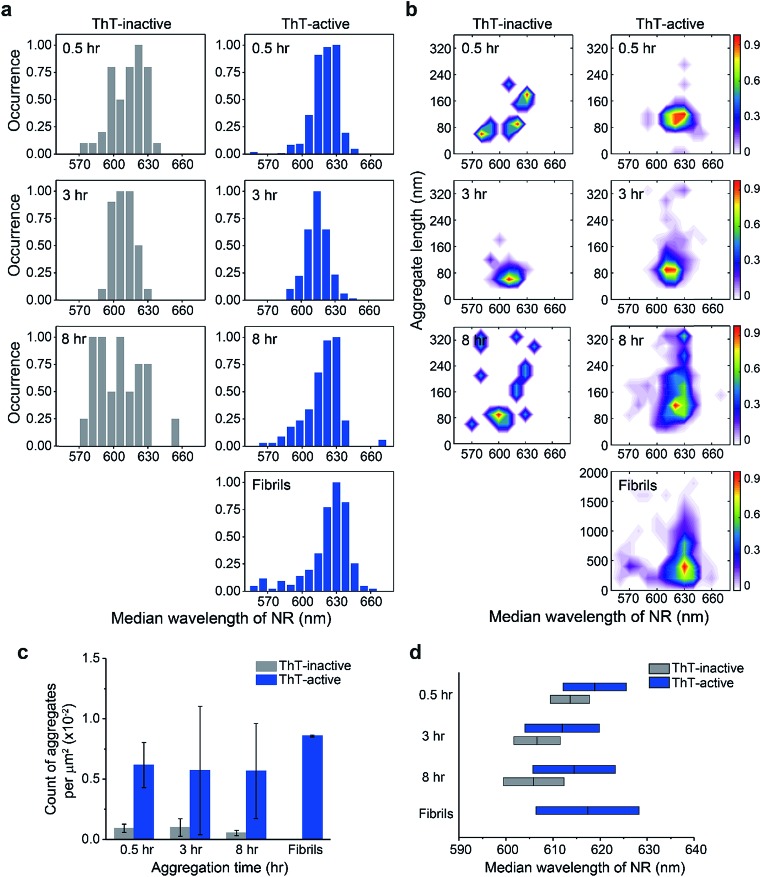
(a) Hydrophobicity distribution of ThT-inactive and ThT-active PrP species at different time points. The median wavelength of Nile red (NR) fluorescence derived from all binding events to a single PrP aggregate was determined to measure the hydrophobicity of individual aggregates. (b) Hydrophobicity landscapes of individual PrP aggregates plotted as a function of their length. The landscape plots are colored by the population density of the aggregates. The distributions shown correspond to the accumulation of three independent measurements. (c) Number of aggregates identified from sPAINT images as a function of time. (d) Median wavelength of NR fluorescence for individual aggregates as a function of time. The bars represent mean values and standard deviations from three independent experiments. The maximum value in each distribution was normalized to 1. The overall number of analyzed PrP aggregates is as follows. At 0.5 h: *N*_ThT-inactive_ = 44, *N*_ThT-active_ = 403; at 3 h: *N*_ThT-inactive_ = 36, *N*_ThT-active_ = 199; at 8 h: *N*_ThT-inactive_ = 24, *N*_ThT-active_ = 261; for fibrils at 48 h: *N*_ThT-active_ = 341.

At early times, only small-sized PrP aggregates were detected using sPAINT with NR dyes (Fig. 1a). No fibrillar species were found at early times, which is consistent with the data as shown in Fig. S1.† Using the super-resolved images, we measured the length of individual aggregates for ThT-inactive and ThT-active species and obtained their length distributions. ThT-active species showed a gradual increase in their length with time, while the length of ThT-inactive species also slightly increased (Fig. 1b), despite their low number (Fig. 2c). The mature fibrils collected at 48 h of aggregation showed a diverse range of length distribution with an average of approximately 550 nm.

Using the median wavelength of NR fluorescence of individual PrP aggregates, we also measured the surface hydrophobicity of these aggregates at different time points for the ThT-inactive and ThT-active species (Fig. 2a and d). The surface of ThT-inactive species was found to be more hydrophobic compared to that of ThT-active species at each time point. In addition, the surface hydrophobicity of the ThT-inactive species gradually increased with time, which suggested a structural reorganization occurred during PrP aggregation. However, the ThT-inactive species only constituted a small fraction of all aggregates (Fig. 2c). In contrast, the dominant ThT-active aggregates showed no clear changes in hydrophobicity with time. To gain more insights into the aggregate conformations, we plotted the hydrophobicity landscapes of individual aggregates against their length (Fig. 2b). ThT-active PrP aggregates were shown to grow in size with similar surface hydrophobicity at the early stages of aggregation.

**Fig. 3:**
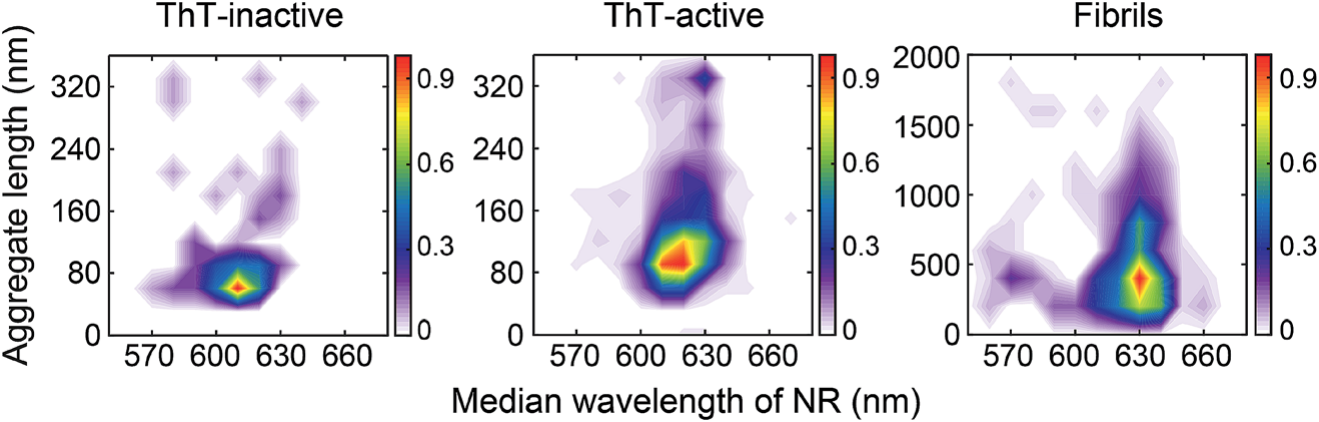
Accumulated hydrophobicity landscapes of ThT-inactive and ThT-active PrP species over all time points compared to mature fibrils formed after 48 h aggregation. The landscape plots are colored by the population density of the aggregates. The overall number of analyzed PrP aggregates is as follows: *N*_ThT-inactive_ = 103, *N*_ThT-active_ = 863, *N*_ThT-active_ for fibrils at 48 h = 341.

The difference between the surface structure of ThT-inactive and ThT-active aggregates was more clearly visualized by combining the hydrophobicity landscapes from 0.5, 3, to 8 h (Fig. 3). While ThT-inactive aggregates were generally less than 80 nm in length, ThT-active aggregates were longer with a wider range of lengths, and their hydrophobicity was similar to that of mature fibrils collected at 48 h. This suggests that ThT-active aggregates were structurally more similar to mature fibrils.

The total number of PrP aggregates detected using sPAINT showed only small changes with time (Fig. 2c). This is consistent with TIRF data (Fig. S2b†), which suggested that only a small fraction of PrP aggregates that formed at early stages of the aggregation ultimately grow into mature fibrils. We then further analyzed the ThT intensity of individual ThT-active aggregates from the TIRF data. The ThT intensity distributions showed the presence of two populations, high-intensity (H species, peak at ∼15 a.u.) and low-intensity species (L species, peak at <1 a.u.) (Fig. S2c†). It has been previously shown that the H species appeared to be larger in size with a molecular weight of >300 kDa (*i.e.* >12 PrP molecules), while the L species was <300 kDa.^35^ These results indicate that the early-formed PrP aggregates observed are mainly small-sized oligomers. It has to be noted that long fibrils were not efficiently detected in our TIRF imaging system, possibly due to the structural fragility of PrP fibrils. However, these experiments provide structural insights into early-formed oligomers based on ThT intensity of individual aggregates and their PK resistance as discussed below.

### PrP oligomers undergo a PK-sensitive to PK-resistant structural conversion

**Fig. 4:**
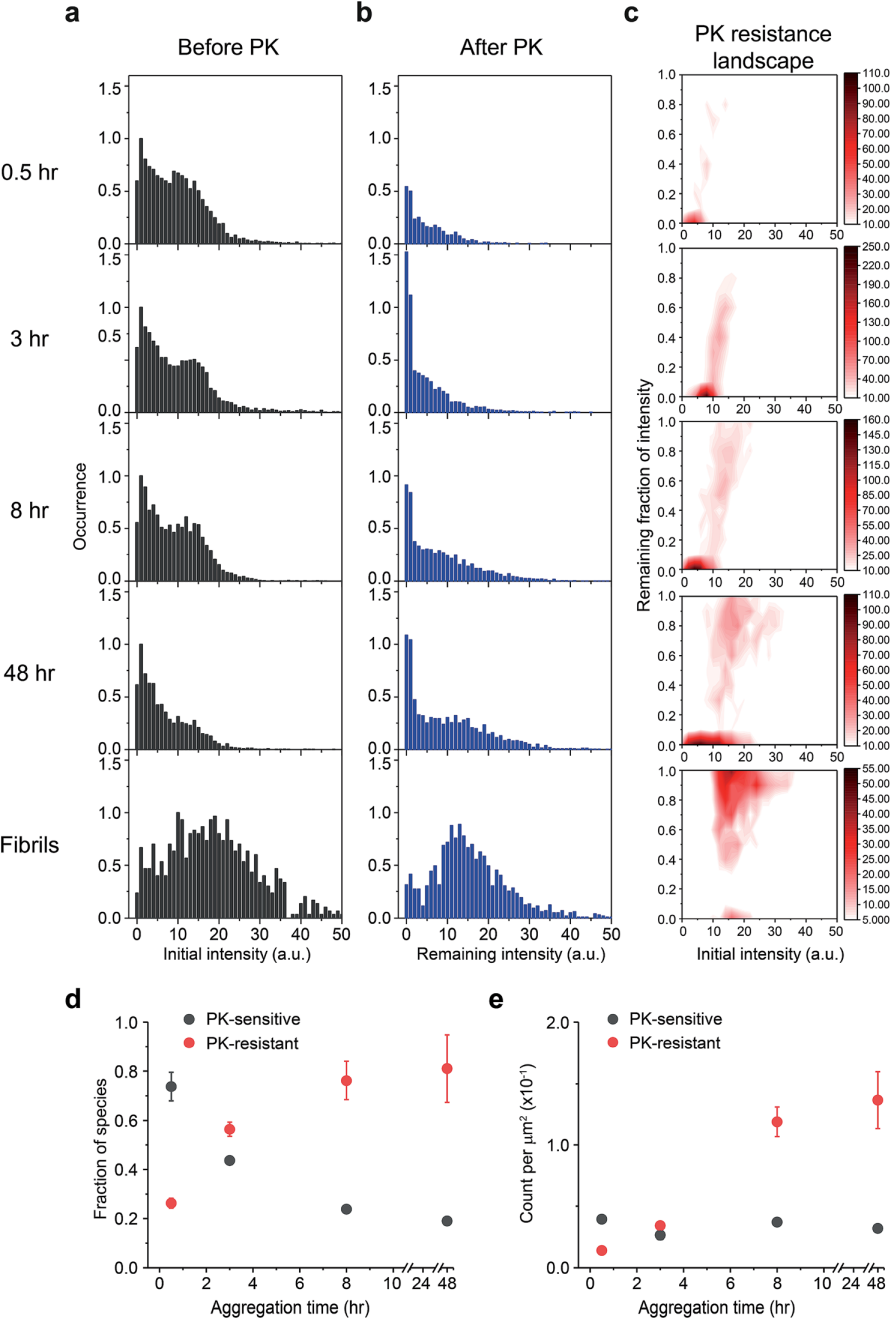
Time-dependent increase of Proteinase K (PK) resistance during PrP aggregation in the presence of 2 M GdnHCl at 37  °C with shaking at 200 rpm. The ThT intensity distributions of PrP aggregates at different time points (a) before and (b) after PK treatment. PK was added at different times to the glass surface that contained the PrP aggregates and the slide chamber sealed to prevent fluid evaporation. The change in ThT intensity of individual particles was followed by continual imaging with the fixed field of views at 37 °C incubation. Normalized PK resistance was calculated as the fraction of the ThT intensity after 1 h proteolytic digestion compared to that seen at the start of the measurement. (c) The PK resistance of individual aggregate plotted against their initial intensity. The plots shown correspond to the combined results of three independent measurements. The PK resistance landscape plots were globally fitted to 2D-Gaussian functions to estimate the fraction of PK-sen and PK-res species. Change in (d) the fraction and (e) the number of PK-sen and PK-res species of PrP aggregates were then plotted as a function of time. The error bars represent standard deviations from three independent experiments.

Next, we examined the PK susceptibility of the ThT-active species as a function of time. The decrease of ThT intensity of individual aggregates at defined time points was measured after 1 h-proteolytic digestion (Fig. 4a–c). Most of the ThT-active oligomers were initially PK-sensitive (PK-sen), and PK-resistant species (PK-res) developed over time (Fig. 4c). The initial ThT intensity of the aggregates before PK treatment increased over time, suggesting the molecular size of PrP aggregates increased over time, which is consistent with the data shown in Fig. 1 and S2b†. Interestingly, the relationship between PK resistance and the initial ThT intensity also suggested that the H species of PrP aggregates were comprised of both PK-sen and PK-res species (Fig. 4c). Next, we carried out 2D-Gaussian fitting of the PK resistance data at different time points and acquired the fraction and the number of the PK-sen/PK-res species (Fig. 4d and e). Since the increase of the fraction of the PK-res species was at the same rate as that of the decrease of the PK-sen species, it suggested that there was a direct structural conversion from the PK-sen to PK-res conformation.

To gain more insight between the correlation of PK resistance and ThT intensity, we combined the PK resistance data (Fig. 4d) with the ThT intensity distributions (Fig. S2c†). The PK-sen and PK-res oligomers could be further sub-classified based on their ThT intensity, either L or H. Therefore, four oligomeric species were found: (1) PK-sen/low-intensity (S_L_); (2) PK-sen/high-intensity (S_H_); (3) PK-res/low-intensity (R_L_); and (4) PK-res/high-intensity (R_H_). Combining their kinetic data, we showed that at the early stage of PrP aggregation, the number of R_L_ and R_H_ increased with time, in contrast to S_L_ and S_H_, which remained unchanged at a low level (Fig. S2d†).

### PK-sensitive oligomers are more capable of disrupting the lipid membrane than fibrils

The oligomeric aggregates of PrP have been shown to be more toxic than fibrils both *in vitro* and *in vivo*.^28^,^36^,^37^ From biophysical studies and computer simulations on other aggregated proteins,^13^,^38^–^44^ the origin of the cytotoxicity is suggested to be non-specific membrane disruption. This partially permeabilizes the lipid membrane of cells, resulting in Ca^2+^ influx and the disruption of cellular homeostasis.^13^,^15^,^39^,^42^,^43^,^45^,^46^ To study the potential damaging effect of protein aggregates on lipid membranes, we have recently developed an assay to quantify the ability of aggregates to permeabilize membranes, by measuring the influx of external Ca^2+^ ions with a liposome-encapsulated Ca^2+^-binding dye.^47^ Using this approach, we have quantified the membrane disruption of toxic aggregates of Aβ^43^ and α-synuclein,^48^ tau,^49^ as well as those in human cerebrospinal fluid (CSF) from Alzheimer's disease patients.^50^

**Fig. 5:**
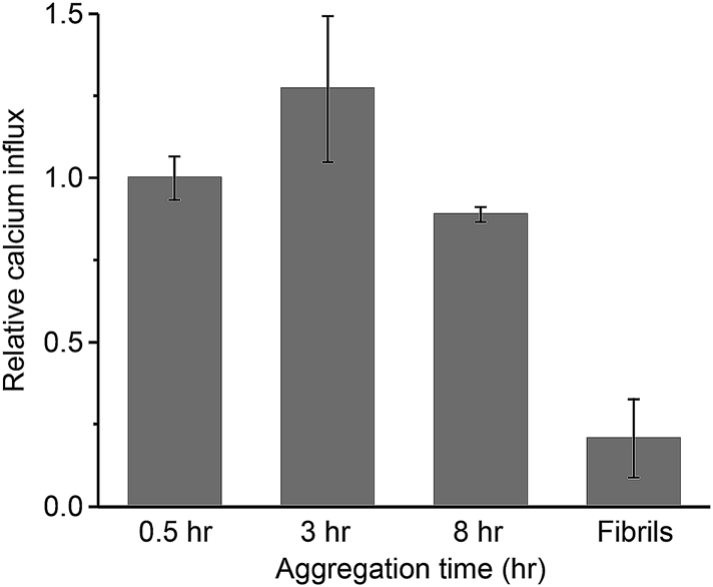
Membrane permeability per PrP aggregate as a function of time. Monomeric PrP was aggregated at a concentration of 27.5 μM in the presence of 2 M GdnHCl at 37  °C with shaking at 200 rpm. At each time point, an aliquot was taken, diluted to a final concentration of 50 nM, and loaded onto a liposome-attached slide surface. The fibrils were collected at 48 h by centrifugation. The increase of Cal-520 (Ca^2+^-binding dye) fluorescence was determined as Ca^2+^ influx and was calibrated with blank background and ionomycin control as described in the Methods. The Ca^2+^ influx from individual experiments was then normalized with the number of ThT-active PrP aggregates observed from TIRF images. The relative influx level at 0.5 h was set as 1. The error bars represent standard deviations from three independent experiments.

We applied PrP aggregates at defined time points onto liposomes that attach to a coverslip surface and then measured the membrane permeabilization induced by the aggregates. In Fig. 5, the membrane permeabilization was normalized by the average number of the aggregates observed in the TIRF images. For the mature fibrils collected after 48 h of aggregation, the membrane permeabilization per aggregate was found to be 4-fold lower than the small-sized aggregates formed at early stages of aggregation. This suggested the inefficiency of PrP fibrils in permeabilizing lipid membranes. In contrast, the early-formed aggregates saw a constant high capability of membrane permeabilization with little change at early aggregation times. This suggested that the PK-res species (R_L_ and R_H_) were less likely to be responsible for the disruption of lipid membranes, as their number increased dramatically over time (Fig. 4e). Instead, the two types of PK-sen species (S_L_ and S_H_) and ThT-inactive species, which all maintained constant numbers over time (Fig. 2c and 4e), were more likely to be the main cause of membrane permeabilization.

## Discussion

The study of the spatial distribution and temporal evolution of the infectious and toxic PrP species is important and requires direct studies of infectivity and toxicity and identification of the species responsible. However, physical characterization of the PrP species has proved to be technically challenging. Single-aggregate imaging methods provide an *in vitro* approach to characterize the various species of PrP aggregates. We have explored the aggregation kinetics of recombinant PrP at early stages under partially denaturing conditions. According to our measurements, five oligomeric species with distinct structural characteristics have been identified: (1) PK-sen/low-intensity oligomers (S_L_); (2) PK-sen/high-intensity oligomers (S_H_); (3) PK-res/low-intensity oligomers (R_L_); (4) PK-res/high-intensity oligomers (R_H_); and (5) ThT-inactive oligomers. The presence of S_L_, S_H_, and ThT-inactive oligomers at early times and their constant numbers during aggregation suggests that they are in kinetic equilibrium with monomers. In contrast, from the temporal change of PK-sen and PK-res species in Fig. 4d, it is suggested that S_L_ and S_H_ undergo a structural conversion to R_L_ and R_H_, independently, despite the L and H species appearing to have a similar kinetic behavior at early aggregation times within 8 h (Fig. S2c and d†).

**Fig. 6:**
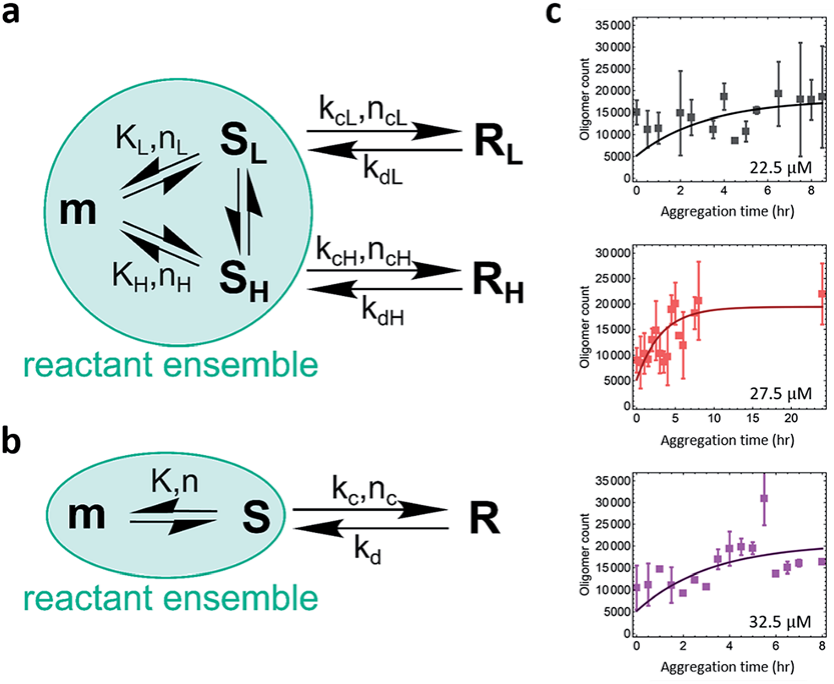
Modeling the kinetics of PrP aggregation. (a) The kinetic model considers the nucleation of PK-sensitive species (S) from the monomer (m) is in equilibrium, and a structural conversion reaction happens between oligomeric populations. (b) A simplified kinetic model that considers a single S → R population, as L and H share similar kinetic parameters. The S species includes S_L_ (low-intensity) and S_H_ (high-intensity), while the R species includes R_L_ (low-intensity) and R_H_ (high-intensity). (c) Global fits of the kinetic profiles of PrP aggregation using the nucleation–dissociation–conversion model with a single S → R population. *K*, equilibrium constant; *n*, reaction order of nucleation; *k*_c_, rate constant of conversion from S to R; *n*_c_, reaction order of conversion; *k*_d_, rate constant of reverse reaction of conversion. See Methods and materials for the derivation of the model.

The quantitative TIRF data provided structural information of ThT-active PrP aggregates with high temporal resolution. Kinetic analysis is an important approach that can be used to determine the microscopic mechanism of the aggregation reaction. The data were analyzed by fitting to a kinetic model for protein aggregation^15^,^51^–^53^ (Fig. 6; see Methods and materials for the derivation of the model). Compared to the previous model for α-synuclein aggregation,^53^ the kinetics for PrP aggregation (Fig. 6a) contains reversible reactions that cannot be neglected and follows a nucleation–dissociation–conversion model. This is similar to the previous work with the yeast prion Ure2, which dissociates back to monomers during the initial nucleation process.^29^ In the kinetic model for the early stage of PrP aggregation, S_L_ and S_H_ share similar kinetic parameters, as do R_L_ and R_H_ (Table 1). This suggests that the L and H species are likely to interconvert on the time scale of the measurements. Therefore for convenience, the model can be simplified to that shown in Fig. 6b, where S_L_ and S_H_ are treated as a single species, as are R_L_ and R_H_. Based on this simplified model, the fitted kinetic parameters are shown in Table 2. This means the half-life for PK-sen → PK-res conversion is roughly 1 hour under our aggregation conditions. Our previous measurement for α-synuclein^15^ gave the half-life for the PK-sen → PK-res conversion as about 36 hours, which is slower than that for PrP by an order of magnitude. The fast conversion rate for PrP may be partially due to the semi-denaturing condition, but still, it suggests that the conversion rate is fundamentally faster in the case of PrP.

**Table 1:** Fitted parameters for the kinetic model of PrP aggregation. In this model, the L and H species are nucleated from monomers and have independent aggregation reactions (*i.e.* S_L_ → R_L_; S_H_ → R_H_). *K*, equilibrium constant; *n*, reaction order of nucleation; *k*_c_, rate constant of PK-sen → PK-res conversion; *n*_c_, reaction order of conversion; *k*_d_, rate constant of reverse reaction of conversion

	*K* (count per μM)	*n*	*k*_c_ (h^–1^)	*n*_c_	*k*_d_ (h^–1^)
L	120	1	0.80	0	0.40
H	69	1	0.93	0	0.23

**Table 2:** Fitted parameters for the simplified kinetic model of PrP aggregation. In the simplified model, the L and H species are treated as a single species due to their similar kinetics. The PK-sensitive species is formed by monomer nucleation and then converts to PK-resistant species. *K*, equilibrium constant; *n*, reaction order of nucleation; *k*_c_, rate constant of PK-sen → PK-res conversion; *n*_c_, reaction order of conversion; *k*_d_, rate constant of reverse reaction of conversion

	*K* (count per μM)	*n*	*k*_c_ (h^–1^)	*n*_c_	*k*_d_ (h^–1^)
Total (L + H)	189	1	0.77	0	0.27

It is not surprising that in the absence of a PrP^Sc^ template, the aggregation of recombinant PrP results in a range of abnormal β-sheet-rich isoforms. Many biochemical studies have been carried out to generate and characterize PrP oligomers *in vitro*, although these oligomers were often obtained under variable aggregation conditions and from different versions of PrP. Two types of PrP oligomers have been shown to be kinetically stable and do not form fibrils.^16^–^20^ This may be due to recombinant PrP that is trapped in kinetic local maxima during the unfolding/refolding steps. PrP oligomerization has previously been followed either on a mica or gold surface.^54^–^56^ However, it is unclear if the oligomers formed on a hydrophilic surface in these experiments eventually form fibrils or show similar structural characteristics to oligomers obtained in other studies. Furthermore, the use of truncated versions of PrP and the lack of co-factors may also contribute to increasing the chance of being trapped in local maxima. In the present study, full-length PrP aggregation has been carried out under a condition that favors the formation of amyloid fibrils. Therefore, at least a fraction of the ThT-active oligomers observed is likely to be on-pathway intermediates to fibril formation. Considering the ThT-inactive species, a previous study reports that they are off the pathway to fibrils.^57^ This is different from current findings, where we observe that the ThT-inactive species undergo a structural transition to a more hydrophobic conformation. Our observation of an unchanged number of the ThT-inactive species suggests that they may be at equilibrium with other aggregate species, some of which undergo a structural conversion and continue to form fibrils.

The small changes in the number of total oligomeric populations formed at the early stage of aggregation suggest that many of them stay in the soluble state and can dissociate back to monomers. It is likely that only a minor fraction of the PK-res oligomers grow into mature fibrils. Apart from the molecular size (based on the observation from ThT intensity distribution), it is difficult to distinguish them using other structural approaches, such as surface hydrophobicity or PK resistance, indicating that L and H species have similar conformations. Despite the L and H species showing similar kinetics, the fraction of the H species decreased at 48 h when fibril formation reached a plateau (Fig. S2c†), suggesting that it is the H species (>300 kDa), that ultimately grows to mature fibrils. Studies of the purified hamster PrP^Sc^ support this concept since its oligomeric forms appear to form two species with different molecular sizes. The molecular weight of the most infectious PrP aggregates is found to be ∼300 to 600 kDa (comprising 14–28 PrP molecules),^25^,^27^ while the small oligomeric species is ∼100 to 150 kDa (comprising 4–6 PrP molecules) and is not infectious.^27^,^58^

Detailed elucidation of the ultrastructure of PrP^Sc^ has been hindered largely due to the difficulties in obtaining sufficient amounts of this aggregated species. Several structures for PrP^Sc^ have been proposed based on a variety of different experimental approaches.^59^–^66^ There are two main competing models where the structure of PrP^Sc^ contains either a parallel in-register intermolecular β-sheet (PIRIBS)^62^,^67^ or a β-solenoid^66^,^68^ architecture. Based on the current data, we can discuss the structural properties of our PrP aggregates in the context of these two models. It is important to note that our recombinant PrP aggregates were obtained using semi-denaturing conditions, similar to those used for generating PrP fibrils with a parallel in-register structure.^32^ Compared to PrP^C^, PrP^Sc^ is usually partially resistant to PK digestion in the C-terminal portion of the molecule, indicating the existence of a highly stable inter-molecular interaction within a single aggregate.^69^ PK resistance of PrP^Sc^ has been shown to be strongly dependent on the quaternary structure of PrP^Sc^.^25^,^70^ The increase of PK resistance in Fig. 4 suggests that recombinant PrP adopts a more compact high-ordered structure during the formation of fibrils. This is unlikely to be due to the increased molecular size, because we did not see a correlation between PK resistance and the accumulation of L/H oligomers. From Fig. 2, the surface hydrophobicity of the ThT-active aggregates does not show a clear change over time and is similar to that of mature fibrils. This indicates that despite the increase of PK resistance, the exposed regions of individual monomers in the fibrils is similar to that in early-formed oligomers. The oligomers are unlikely to have the compact β-solenoid structure,^66^,^71^ since this has less exposed residues and is expected to be highly PK-resistant. In contrast, the parallel in-register model^62^,^67^ has extended loops and hairpins exposed to solvent molecules. Therefore, if the PrP oligomers adopt a parallel in-register structure during the transition to fibrils, the surface hydrophobicity would remain constant, while PK resistance would increase due to the increase of the inter-molecular interaction between monomers in the aggregate, which is consistent with our results.

Although the origin of the cytotoxicity induced by aggregated proteins might be complicated, it appears to be highly correlated to the ability of the aggregates to disrupt lipid membranes of cellular components. The membrane permeability assay represents a means to quantitatively measure the ability of PrP aggregates to permeabilize lipid membranes and to determine the most effective species. Our data show that early-formed PrP oligomers possess higher membrane permeability than mature fibrils and that the ThT-inactive or PK-sen oligomers are likely to be responsible for inducing calcium influx in membranes. This is consistent with previous findings that toxic oligomers are structurally loosely-packed^39^ and that they may result from PK-sen species.^22^ It also suggests that these toxic species of PrP aggregates may include ThT-inactive species, which are present at a low level, relatively small in size, and technically difficult to detect. Therefore, despite the fact that the current measurement is based on *in vitro* aggregation of PrP which does not involve co-factors and translational modifications, this work provides important insights into the complexity of PrP aggregation at early stages of aggregation and the structure of the aggregates formed. However, the difference between bona fide prions and the currently observed PrP oligomers remains to be elucidated.

Overall, our work reveals that at least five types of aggregates can co-exist during PrP aggregation. The ThT-inactive oligomers and PK-sen oligomers remain at a constant number over time and are better at disrupting lipid membranes and inducing Ca^2+^ influx. In contrast, PK-res oligomers are likely formed by a structural conversion from the PK-sen species and are likely to form fibrils. According to ThT intensity, the structurally different PK-sen/PK-res species can be sub-divided into the L and H species, which are different in size and yet share a similar kinetic behavior. Therefore, PrP amplification and lipid membrane disruption are likely mediated by different aggregate species with distinct structural properties. This study provides insights into prion diversity and why some protein aggregates can act as efficient pathogens whereas others cannot. Despite requiring further studies *in vivo*, these aggregate species identified can be specific targets for therapeutic intervention. Therefore, reducing the population of the specific oligomeric species or promoting their removal pathways can be potentially important for inhibiting the aggregation process of PrP propagation.

## Conflicts of interest

There are no conflicts to declare.

## Supplementary Material

Supplementary information

## References

[cit1] Aguzzi A., Nuvolone M., Zhu C. (2013). Nat. Rev. Immunol..

[cit2] Prusiner S. B. (1982). Science.

[cit3] Liberski P. P., Brown P., Xiao S. Y., Gajdusek D. C. (1991). J. Comp. Pathol..

[cit4] DeArmond S. J., McKinley M. P., Barry R. A., Braunfeld M. B., McColloch J. R., Prusiner S. B. (1985). Cell.

[cit5] McKinley M. P., Bolton D. C., Prusiner S. B. (1983). Cell.

[cit6] Bucciantini M., Calloni G., Chiti F., Formigli L., Nosi D., Dobson C. M., Stefani M. (2004). J. Biol. Chem..

[cit7] Bucciantini M., Giannoni E., Chiti F., Baroni F., Formigli L., Zurdo J., Taddei N., Ramponi G., Dobson C. M., Stefani M. (2002). Nature.

[cit8] Nath S., Agholme L., Kurudenkandy F. R., Granseth B., Marcusson J., Hallbeck M. (2012). J. Neurosci..

[cit9] Bemporad F., Chiti F. (2012). Chem. Biol..

[cit10] Chen S. W., Drakulic S., Deas E., Ouberai M., Aprile F. A., Arranz R., Ness S., Roodveldt C., Guilliams T., De-Genst E. J., Klenerman D., Wood N. W., Knowles T. P. J., Alfonso C., Rivas G., Abramov A. Y., Valpuesta J. M., Dobson C. M., Cremades N. (2015). Proc. Natl. Acad. Sci. U. S. A..

[cit11] Ruggeri F. S., Longo G., Faggiano S., Lipiec E., Pastore A., Dietler G. (2015). Nat. Commun..

[cit12] Jucker M., Walker L. C. (2013). Nature.

[cit13] Knowles T. P. J., Vendruscolo M., Dobson C. M. (2014). Nat. Rev. Mol. Cell Biol..

[cit14] Serio T. R., Cashikar A. G., Kowal A. S., Sawicki G. J., Moslehi J. J., Serpell L., Arnsdorf M. F., Lindquist S. L. (2000). Science.

[cit15] Cremades N., Cohen S. I. A., Deas E., Abramov A. Y., Chen A. Y., Orte A., Sandal M., Clarke R. W., Dunne P., Aprile F. A., Bertoncini C. W., Wood N. W., Knowles T. P. J., Dobson C. M., Klenerman D. (2012). Cell.

[cit16] Bocharova O. V., Breydo L., Parfenov A. S., Salnikov V. V., Baskakov I. V. (2005). J. Mol. Biol..

[cit17] Legname G., Baskakov I. V., Nguyen H.-O. B., Riesner D., Cohen F. E., DeArmond S. J., Prusiner S. B. (2004). Science.

[cit18] Jackson G. S., Hosszu L. L., Power A., Hill A. F., Kenney J., Saibil H., Craven C. J., Waltho J. P., Clarke A. R., Collinge J. (1999). Science.

[cit19] Jackson G. S., Hill A. F., Joseph C., Hosszu L., Power A., Waltho J. P., Clarke A. R., Collinge J. (1999). Biochim. Biophys. Acta.

[cit20] Makarava N., Ostapchenko V. G., Savtchenko R., Baskakov I. V. (2009). J. Biol. Chem..

[cit21] Trevitt C. R., Hosszu L. L. P., Batchelor M., Panico S., Terry C., Nicoll A. J., Risse E., aTaylor W., Sandberg M. K., Al-Doujaily H., Linehan J. M., Saibil H. R., Scott D. J., Collinge J., Waltho J. P., Clarke A. R. (2014). J. Biol. Chem..

[cit22] Sandberg M. K., Al-Doujaily H., Sharps B., DeOliveira M. W., Schmidt C., Richard-Londt A., Lyall S., Linehan J. M., Brandner S., Wadsworth J. D. F., Clarke A. R., Collinge J. (2014). Nat. Commun..

[cit23] Colby D. W., Wain R., Baskakov I. V., Legname G., Palmer C. G., Nguyen H.-O. B., Lemus A., Cohen F. E., DeArmond S. J., Prusiner S. B. (2010). PLoS Pathog..

[cit24] Cronier S., Gros N., Tattum M. H., Jackson G. S., Clarke A. R., Collinge J., Wadsworth J. D. F. (2008). Biochem. J..

[cit25] Tzaban S., Friedlander G., Schonberger O., Horonchik L., Yedidia Y., Shaked G., Gabizon R., Taraboulos A. (2002). Biochemistry.

[cit26] Safar J., Wille H., Itri V., Groth D., Serban H., Torchia M., Cohen F. E., Prusiner S. B. (1998). Neonat. Med..

[cit27] Silveira J. R., Raymond G. J., Hughson A. G., Race R. E., Sim V. L., Hayes S. F., Caughey B. (2005). Nature.

[cit28] Simoneau S., Rezaei H., Salès N., Kaiser-Schulz G., Lefebvre-Roque M., Vidal C., Fournier J.-G., Comte J., Wopfner F., Grosclaude J., Schätzl H., Lasmézas C. I. (2007). PLoS Pathog..

[cit29] Yang J., Dear A. J., Michaels T. C. T., Dobson C. M., Knowles T. P. J., Wu S., Perrett S. (2018). J. Am. Chem. Soc..

[cit30] Sandberg M. K., Al-Doujaily H., Sharps B., Clarke A. R., Collinge J. (2011). Nature.

[cit31] Horrocks M. H., Lee S. F., Gandhi S., Magdalinou N. K., Chen S. W., Devine M. J., Tosatto L., Kjaergaard M., Beckwith J. S., Zetterberg H., Iljina M., Cremades N., Dobson C. M., Wood N. W., Klenerman D. (2016). ACS Chem. Neurosci..

[cit32] Dutta A., Chen S., Surewicz W. K. (2013). FEBS Lett..

[cit33] Bongiovanni M. N., Godet J., Horrocks M. H., Tosatto L., Carr A. R., Wirthensohn D. C., Ranasinghe R. T., Lee J.-E., Ponjavic A., Fritz J. V., Dobson C. M., Klenerman D., Lee S. F. (2016). Nat. Commun..

[cit34] Lee J.-E., Sang J. C., Rodrigues M., Carr A. R., Horrocks M. H., De S., Bongiovanni M. N., Flagmeier P., Dobson C. M., Wales D. J., Lee S. F., Klenerman D. (2018). Nano Lett..

[cit35] Sang J. C., Meisl G., Thackray A. M., Hong L., Ponjavic A., Knowles T. P. J., Bujdoso R., Klenerman D. (2018). J. Am. Chem. Soc..

[cit36] Kazlauskaite J., Young A., Gardner C. E., Macpherson J. V., Vénien-Bryan C., Pinheiro T. J. T. (2005). Biochem. Biophys. Res. Commun..

[cit37] Novitskaya V., Bocharova O. V., Bronstein I., Baskakov I. V. (2006). J. Biol. Chem..

[cit38] Cheon M., Chang I., Mohanty S., Luheshi L. M., Dobson C. M., Vendruscolo M., Favrin G. (2007). PLoS Comput. Biol..

[cit39] Campioni S., Mannini B., Zampagni M., Pensalfini A., Parrini C., Evangelisti E., Relini A., Stefani M., Dobson C. M., Cecchi C., Chiti F. (2010). Nat. Chem. Biol..

[cit40] Narayan P., Ganzinger K. A., McColl J., Weimann L., Meehan S., Qamar S., Carver J. A., Wilson M. R., St George-Hyslop P., Dobson C. M., Klenerman D. (2013). J. Am. Chem. Soc..

[cit41] Serra-Batiste M., Ninot-Pedrosa M., Bayoumi M., Gairí M., Maglia G., Carulla N. (2016). Proc. Natl. Acad. Sci. U. S. A..

[cit42] Andreasen M., Lorenzen N., Otzen D. (2015). Biochim. Biophys. Acta.

[cit43] Benilova I., Karran E., DeStrooper B. (2012). Nat. Neurosci..

[cit44] Soto C. (2003). Nat. Rev. Neurosci..

[cit45] Evangelisti E., Cascella R., Becatti M., Marrazza G., Dobson C. M., Chiti F., Stefani M., Cecchi C. (2016). Sci. Rep..

[cit46] Haass C., Selkoe D. J. (2007). Nat. Rev. Mol. Cell Biol..

[cit47] Flagmeier P., De S., Wirthensohn D. C., Lee S. F., Vincke C., Muyldermans S., Knowles T. P. J., Gandhi S., Dobson C. M., Klenerman D. (2017). Angew. Chem., Int. Ed..

[cit48] Varela J. A., Rodrigues M., De S., Flagmeier P., Gandhi S., Dobson C. M., Klenerman D., Lee S. F. (2018). Angew. Chem., Int. Ed..

[cit49] Kundel F., De S., Flagmeier P., Horrocks M. H., Kjaergaard M., Shammas S. L., Jackson S. E., Dobson C. M., Klenerman D. (2018). ACS Chem. Biol..

[cit50] Drews A., De S., Flagmeier P., Wirthensohn D. C., Chen W.-H., Whiten D. R., Rodrigues M., Vincke C., Muyldermans S., Paterson R. W., Slattery C. F., Fox N. C., Schott J. M., Zetterberg H., Dobson C. M., Gandhi S., Klenerman D. (2017). Cell Rep..

[cit51] Tosatto L., Horrocks M. H., Dear A. J., Knowles T. P. J., Dalla Serra M., Cremades N., Dobson C. M., Klenerman D. (2015). Sci. Rep..

[cit52] Horrocks M. H., Tosatto L., Dear A. J., Garcia G. A., Iljina M., Cremades N., Dalla Serra M., Knowles T. P. J., Dobson C. M., Klenerman D. (2015). Anal. Chem..

[cit53] Iljina M., Garcia G. A., Horrocks M. H., Tosatto L., Choi M. L., Ganzinger K. A., Abramov A. Y., Gandhi S., Wood N. W., Cremades N., Dobson C. M., Knowles T. P. J., Klenerman D. (2016). Proc. Natl. Acad. Sci. U. S. A..

[cit54] Wang B., Guo C., Lou Z., Xu B. (2015). Chem. Commun..

[cit55] Lou Z., Wang B., Guo C., Wang K., Zhang H., Xu B. (2015). Colloids Surf., B.

[cit56] Pan Y., Wang B., Zhang T., Zhang Y., Wang H., Xu B. (2016). Chem. Commun..

[cit57] Baskakov I. V., Legname G., Baldwin M. a., Prusiner S. B., Cohen F. E. (2002). J. Biol. Chem..

[cit58] Riesner D., Kellings K., Post K., Wille H., Serban H., Groth D., Baldwin M. A., Prusiner S. B. (1996). J. Virol..

[cit59] Wille H., Michelitsch M. D., Guenebaut V., Supattapone S., Serban A., Cohen F. E., Agard D. a., Prusiner S. B. (2002). Proc. Natl. Acad. Sci. U. S. A..

[cit60] Govaerts C., Wille H., Prusiner S. B., Cohen F. E. (2004). Proc. Natl. Acad. Sci. U. S. A..

[cit61] DeMarco M. L., Daggett V. (2004). Proc. Natl. Acad. Sci. U. S. A..

[cit62] Cobb N. J., Sönnichsen F. D., McHaourab H., Surewicz W. K. (2007). Proc. Natl. Acad. Sci. U. S. A..

[cit63] Hafner-Bratkovic I., Bester R., Pristovsek P., Gaedtke L., Veranic P., Gaspersic J., Mancek-Keber M., Avbelj M., Polymenidou M., Julius C., Aguzzi A., Vorberg I., Jerala R. (2011). J. Biol. Chem..

[cit64] Knaus K. J., Morillas M., Swietnicki W., Malone M., Surewicz W. K., Yee V. C. (2001). Nat. Struct. Biol..

[cit65] Diaz-Espinoza R., Soto C. (2012). Nat. Struct. Mol. Biol..

[cit66] Vázquez-Fernández E., Vos M. R., Afanasyev P., Cebey L., Sevillano A. M., Vidal E., Rosa I., Renault L., Ramos A., Peters P. J., Fernández J. J., vanHeel M., Young H. S., Requena J. R., Wille H. (2016). PLoS Pathog..

[cit67] Lu X., Wintrode P. L., Surewicz W. K. (2007). Proc. Natl. Acad. Sci. U. S. A..

[cit68] Wille H., Bian W., McDonald M., Kendall A., Colby D. W., Bloch L., Ollesch J., Borovinskiy A. L., Cohen F. E., Prusiner S. B., Stubbs G. (2009). Proc. Natl. Acad. Sci. U. S. A..

[cit69] Prusiner S. B., Scott M. R., DeArmond S. J., Cohen F. E. (1998). Cell.

[cit70] Pastrana M. A., Sajnani G., Onisko B., Castilla J., Morales R., Soto C., Requena J. R. (2006). Biochemistry.

[cit71] Wille H., Requena J. R. (2018). Pathogens.

