# Supplementary material for: Direct observation of prion protein oligomer formation reveals an aggregation mechanism with multiple conformationally distinct species
Source: Chem Sci. 2019 Mar 25;10(17):4588–97. doi: 10.1039/c8sc05627g (PMC6492631; doi:10.1039/c8sc05627g)
Supplement: Supplementary file 1 [file SC-010-C8SC05627G-s001.pdf]

Electronic Supplementary Material (ESI) for Chemical Science.
This journal is © The Royal Society of Chemistry 2019

Direct observation of prion protein oligomer formation reveals an aggregation mechanism with multiple conformationally distinct species

Supporting Information

Jason C. Sang[1], Ji-Eun Lee[1], Alexander J. Dear[1], Suman De[1], Georg Meisl[1], Alana M. Thackray[2], Raymond Bujdoso[2], Tuomas P.J. Knowles[1], David Klenerman[1]*

[1]Department of Chemistry, University of Cambridge, Cambridge, CB2 1EW, UK
[2]Department of Veterinary Medicine, University of Cambridge, CB3 0ES, UK

Table of Contents

Methods and Materials....................2
References....................7
Supplementary Figure 1....................8
Supplementary Figure 2....................9

Methods and Materials

PrP aggregation

Recombinant wild-type mouse PrP (23-231), with an N-terminal methionine and serine residue for optimum bacterial expression[1], was generated as described previously[2]. Amyloid fibril formation was carried out by aggregation under semi-denaturing conditions[3]. PrP solution (1.9 mg/mL) was added to a 1.5 mL centrifugal tube containing the reaction buffer (50 mM sodium phosphate, pH 7.0) and 2 M guanidine hydrochloride (GdnHCl, ≥ 99%, Sigma-Aldrich) to a final concentration of 22.5, 27.5, or 32.5 μM and a volume of 150 μL. The reaction was carried out at 37 °C with 200 rpm shaking in an orbital incubator (ES-20 environmental shaker-incubator, Grant-Bio). To isolate large fibrils in solution, protein aggregates formed after 48 hr, when the number of aggregates reached equilibrium, were centrifuged at 16,000 ×g for 30 min and the pellet was collected.

Fluorescent dye solution and slide preparation

ThT and Nile red (NR) solutions were prepared as previously described[4,5]. ThT (Sigma-Aldrich) stock solution was prepared by fully dissolving the dye in pure dimethyl sulfoxide (DMSO, ≥ 99.9%, Sigma-Aldrich) to a concentration of 10-30 M with vigorous vortexing as a stock solution. The working solution was prepared at a concentration of 50 μM by diluting in the reaction buffer. NR (Thermo Fisher Scientific) stock solution was prepared by dissolving NR into DMSO to a concentration of 1 mM, divided into 10 μL aliquots, flash frozen in liquid N_2, and stored in the dark at -80 °C until required. For the working solution, the NR aliquot was diluted into the reaction buffer to a final concentration of 2 nM. All solutions in this study were filtrated by a 0.02 μm syringe filter (Anotop 10 inorganic membrane filter, Whatman) before use.

Borosilicate glass coverslips (Marienfeld, VWR International, 20×20 mm) were cleaned by argon plasma (PDC-002, Harrick Plasma) for 1 hr, and then attached to Frame-Seal slide chambers (Bio-Rad, 9×9 mm). The surface was coated with poly-L-lysine (PLL, MW = 150,000 - 300,000, 0.01%, Sigma-Aldrich) for 30 min.

Kinetic measurements with TIRF imaging

At each given time point, an aliquot was discarded from the reaction, diluted with the reaction buffer to a final concentration of 300 nM in the presence of 25 μM ThT. The sample mix was added to the coverslip and incubated for 10 minutes allowing aggregates to adhere to the surface of glass coverslips. The coverslip was then immediately loaded onto the microscope stage for imaging.

TIRF imaging was carried out using a home-built total internal reflection fluorescence microscopy (TIRFM) setup as previously described[4]. In brief, a 35 mW diode laser (Oxxius, LBX-LD) with a wavelength of 405 nm was directed into a 60× magnification oil-immersion TIRF objective (ApoN TIRF, Olympus, NA = 1.49) mounted on an inverted microscope (Eclipse Ti-S, Nikon). The fluorescence signal was recorded by an EMCCD camera (Evolve Delta 512, Photometrics) using the open source

microscopy platform Micromanager. For each time point in the kinetic measurements, three random areas that contain a 3×3 grid were recorded with a gap distance of 150 μm (i.e. 9 images were recorded sequentially at adjacent positions in an area; 27 images in total from 3 areas). Each image sequence was acquired as a 100-framed stack at 30 frames s^{-1}.

Individual TIRF images were averaged over all the frames and then subjected to image analysis. Images were analyzed with a custom-written MATLAB script (R2016a, MathWorks) as previously described[4]. For particle identification, images were filtered to remove the modulated background and camera noise. Particles were identified by fine-tuning size and intensity thresholds. To eliminate the background effect in the intensity calculation, the signal-to-background ratio (SBR) was introduced to correct the intensity of pixels, where the SBR is defined as:

$$\frac{\textit{Intensity above the background}}{\textit{Background}}$$

For a given particle, its corrected intensity is the sum of SBR of each pixel within its boundary.

Probing surface hydrophobicity with sPAINT imaging

At defined time points, a protein aliquot was removed and diluted to 50 nM (early-time species) or 100 nM (mature fibrillar species) with the reaction buffer containing 25 μM ThT, 2 nM NR and 10% (v/v) fiducial markers. The sample mix was added to the coverslip and incubated for 10 minutes allowing aggregates to adhere to the surface of glass coverslips, then immediately loaded onto the microscope stage for imaging.

sPAINT imaging was performed using a separate home-built TIRF setup as previously described[5]. In brief, lasers operating at 405 nm continuous wave (CW) diode laser (Cobolt, MLD 0405-06-01-0100-100) for ThT imaging, and 532 nm CW diode-pumped solid-state laser (LASOS Lasertechnik GmbH) for NR imaging were used as excitation sources. The emitted fluorescence was collected through a 60× magnification oil-immersion TIRF objective (Plan Apochromat 60×, NA=1.49, Olympus APON 60XOTIRF) based on an inverted optical microscope (IX73, Olympus). Finally, a mechanical slit (VA100/M, Thorlabs) and a transmission diffraction grating (600 Grooves/mm 22.0° Blaze Angle - GTU13-06, Thorlabs) with a homebuilt grating holder were mounted on the camera port path prior to the EMCCD (Evolve II 512, Photometrics). The fluorescence image was finally projected onto the EMCCD running in frame transfer mode with at 20 Hz, with an electron multiplication gain of 250, operating at -70 °C with a pixel size of 16 μm and automated using the open source microscopy platform Micromanager. Each image sequence was acquired at a frame rate of 20 ms and 200 frames for ThT channel and 2000 frames for NR channel.

The sPAINT images were calibrated as described previously[6]. The distance between 0th and 1st order diffraction patterns are determined by imaging four-color fluorescence beads (0.1 μm in diameter, T7297, Invitrogen) in order to determine the relation to the emission wavelength. Based on the clustered localizations and the final

result images, the spectral information of individual proteins was extracted and plotted using a custom-written MATLAB script (R2014b, Mathworks). A localization threshold of 20 was used to identify individual PrP aggregates. The achievable spatial localization precision of the instrument was measured by imaging diffraction-limited TetraSpeck beads (0.1 mm, T7279, Invitrogen) using a 532 nm excitation laser. Each image was collected as a 500-framed stack at 20 frames s^{-1} at a range of excitation powers and localized using the PeakFit program (GDSC SMLM plugin) in ImageJ (National Institutes of Health, USA). The spectral localization precision was determined by fitting the orange peak of TetraSpeck beads which centers at 581.5 nm.

Single-molecule proteinase K digestion

Proteinase K digestion was performed using TIRF imaging as previously described[4]. Recombinant proteinase K (Fungal, Invitrogen) was prepared at a concentration of 50 μM in the reaction buffer. To determine the PK sensitivity, an aliquot of the protein reaction at defined time points were mixed with vacuum-degassed ThT solution to a final concentration of 300 nM (PrP) and 25 μM (ThT). PK solution was added to a final enzyme concentration of 2 μM prior to imaging. The coverslip was sealed and then loaded on the microscope stage with 37 °C incubation in a custom-made chamber enclosure. The TIRF images were continually acquired at fixed areas for 60 min with a time gap of 2.5 min. Each image contains 100 frames at 30 frames s^{-1}.

Membrane permeability assay using TIRFM

The assay was implemented from our previously described method[7]. Briefly, lipid vesicles were prepared by mixing phospholipids 16:0-18:1 PC (catalog no 850457, Avanti Polar Lipids) and biotinylated lipids 18:1-12:0 biotin-PC (catalog no 860563, Avanti Polar Lipids) at 100:1 ratio in HEPES buffer (pH 6.5). The mean diameter of the vesicles (200 nm) was measured using dynamic light scattering technique Individual vesicles were filled with 100 μM Cal-520 dye (Stratech Scientific Ltd) and non-incorporated free dye molecules were separated from the vesicles using size-exclusion chromatography. Then, glass coverslips were coated with PLL-PEG and biotinylated PLL-PEG (10:1). Lipid vesicles were tethered to the glass coverslips using biotin-neutravidin linkage and incubated with HEPES buffer (pH 6.5). Just before the imaging, HEPES buffer was replaced with 50 μL Ca^{2+}-containing buffer solution L-15. 9 different positions of the coverslips were imaged using an automated BeanShell-based script (as F_{blank}). Then, 50 nM of PrP aggregation solution was added to the coverslips and incubated for 15 minutes. The same area of the coverslips was imaged ($F_{aggregate}$). Then, 10 μL of 1 mg/mL of ionomycin (Cambridge Bioscience Ltd, Cambridge, UK) was added to the same coverslips and the same fields of views were imaged ($F_{ionomycin}$). The average calcium influx was calculated as follows:

$$\mathrm{Ca}^{2+}\mathrm{influx} = \frac{F_{aggregate} - F_{blank}}{F_{ionomycin} - F_{blank}}$$

All the images were taken using a homebuilt TIRF set-up based on an inverted Olympus IX-71 microscope, coupled with 488 nm laser (Toptica, iBeam smart), a 60× magnification oil-immersion TIRF objective (NA=1.49, APON60XO TIRF, Olympus) and an EMCCD camera (Evolve II 512, Photometrics). All the imaging experiments were performed at room temperature (295 K). The acquired images were analyzed using ImageJ to calculate the localized fluorescence intensity of each vesicle and average calcium influx was calculated.

Kinetic analysis

The TIRF data at the early stage of PrP aggregation (≤ 8 hr) was fit to the kinetic model described as follows. Since no fibrils were detected, depletion of monomers could be negligible, and the monomer concentration was approximated to be constant during the modeling process. The number of PK-sen species (S_L and S_H) remained unchanged over the measurement period (Figure S2d). Hence, we modeled the two species as being at equilibrium with monomers over this time period of the measurements. It has to be noted that the constant number of S_L and S_H was very unlikely due to the steady state of the aggregation kinetics, as there was insufficient production of PK-res species or fibrils at the early stage of the aggregation. We therefore approximated the number of the PK-sen species as:

$$S_L = K_L m^{n_L}$$

$$S_H = K_H m^{n_H}$$

where K_L and K_H are the equilibrium constants between monomers and PK-sen species for S_L and S_H, respectively. m is the monomer concentration. n_L and n_H are the equilibrium reaction orders for PK-sen species formation (i.e. they must be ≥ 1). Dissociation of PK-sen species to monomers was assumed to be well-described by a single process with a reaction order of 0.

At the early stage of PrP aggregation, the aggregate length was slightly increased compared to that of fibrils at 48 hr (Figure 1b), and little increase in ThT intensity was seen during this time period (Figure S2c). Furthermore, the molecular size of the H species has been previously shown to be > 300 kDa (i.e. >12 PrP molecules), while the L species was < 300 kDa[4]. Therefore, all of the four ThT-active species: S_L, S_H, R_L, and R_H were highly unlikely to be fibrils. Having established these premises, we were able to explore possible kinetic models for R_L and R_H production.

Because R_L and R_H formed more slowly than S_L and S_H formation (Figure S2d), R_L and R_H could either convert from S_L and S_H, or be nucleated directly from monomers. From the observations, R_L shares a similar ThT intensity profile with S_L (and likewise, in the R_H and S_H pair) (Figure S2c), and the increase of the fraction of the PK-res species was at the same rate as that of the decrease of the PK-sen species (Figure 4d). Therefore, it is highly likely to be the case of a direct PK-sen → PK-res structural conversion (Figure 6a). We therefore described these relations as:

$$\frac{dR_L}{dt} = k_{cL} m^{n_{cL}} S_L - k_{dL} R_L$$

$$\frac{dR_H}{dt} = k_{cH} m^{n_{cH}} S_H - k_{dH} R_H$$

where k_{cL} and k_{cH} are the rate constants of structural conversion of S_L to R_L and of S_H to R_H, respectively. n_{cL} and n_{cH} are the monomer-dependent reaction orders of the conversion reaction. k_{dL} and k_{dH} are the rate constants of depletion of R_L and R_H. Solving the equations, we obtained:

$$R_L = \alpha_L \left(1 - e^{-k_{dL} t}\right)$$

$$R_H = \alpha_H \left(1 - e^{-k_{dH} t}\right)$$

where $\alpha_L = k_{cL} K_L m^{n_{cL}+n_L} / k_{dL}$, and $\alpha_H = k_{cH} K_H m^{(n_{cH}+n_H)} k_{dH}$

To determine the kinetic parameters in this nucleation-dissociation-conversion model, we fitted these equations individually with the quantitative TIRF data obtained from 3 different starting monomer concentrations (Figure S2b), and then we obtained the fitted parameters in Table 1. It has to be noted that our fitted results are only proportional to the monomer concentrations. However, the fitted equilibrium constant *K* may contain unknown proportionality coefficients and thus cannot be compared with other aggregation systems.

From Table 1, S_L and S_H share similar kinetic parameters, as do R_L and R_H. This suggests that the L and H species are likely to interconvert rapidly on the time scale of the measurements. Therefore for convenience, the scheme can be simplified to that shown in Figure 6b, where S_L and S_H are treated as a single species, as are R_L and R_H (Figure 6b). This simplified model is given by:

$$S = Km^n$$

$$R = \alpha\left(1 - e^{-k_d t}\right)$$

where S = S_L + S_H, R= R_L + R_H, and $\alpha = k_c m^{n_c+n} / k_d$. Fitting this simplified model with the number of S and R, we obtained good fits (Figure 6c) with the fitted parameters given in Table 2. It is clear that an equally good fit can be obtained from the two models, which share the same functional form, base on the same TIRF data, as the reaction orders and rate constants are very close or identical between the two models. We note finally that the fitted reaction order of nucleation *n*=1 implies that this rate-determining step involves only 1 monomer for nucleation, which suggested that this process may involve surface interaction e.g. microcentrifugal tube surface.

References

(1) Looman, A. C.; Bodlaender, J.; Comstock, L. J.; Eaton, D.; Jhurani, P.; deBoer, H. A.; vanKnippenberg, P. H.Influence of the Codon Following the AUG Initiation Codon on the Expression of a Modified LacZ Gene in Escherichia Coli. *EMBO J.* **1987**, *6* (8), 2489–2492.

(2) Wong, E.; Thackray, A. M.; Bujdoso, R.Copper Induces Increased β-Sheet Content in the Scrapie-Susceptible Ovine Prion Protein PrPVRQ Compared with the Resistant Allelic Variant PrPARR. *Biochem. J.* **2004**, *380* (1), 273–282.

(3) Dutta, A.; Chen, S.; Surewicz, W. K.The Effect of B2-A2 Loop Mutation on Amyloidogenic Properties of the Prion Protein. *FEBS Lett.* **2013**, *587* (18), 2918–2923.

(4) Sang, J. C.; Meisl, G.; Thackray, A. M.; Hong, L.; Ponjavic, A.; Knowles, T. P. J.; Bujdoso, R.; Klenerman, D.Direct Observation of Murine Prion Protein Replication in Vitro. *J. Am. Chem. Soc.* **2018**, *140* (44), 14789–14798.

(5) Lee, J.-E.; Sang, J. C.; Rodrigues, M.; Carr, A. R.; Horrocks, M. H.; De, S.; Bongiovanni, M. N.; Flagmeier, P.; Dobson, C. M.; Wales, D. J.; Lee, S. F.; Klenerman, D.Mapping Surface Hydrophobicity of α-Synuclein Oligomers at the Nanoscale. *Nano Lett.* **2018**.

(6) Bongiovanni, M. N.; Godet, J.; Horrocks, M. H.; Tosatto, L.; Carr, A. R.; Wirthensohn, D. C.; Ranasinghe, R. T.; Lee, J.-E.; Ponjavic, A.; Fritz, J.V.; Dobson, C. M.; Klenerman, D.; Lee, S. F.Multi-Dimensional Super-Resolution Imaging Enables Surface Hydrophobicity Mapping. *Nat. Commun.* **2016**, *7* (1), 13544.

(7) Flagmeier, P.; De, S.; Wirthensohn, D. C.; Lee, S. F.; Vincke, C.; Muyldermans, S.; Knowles, T. P. J.; Gandhi, S.; Dobson, C. M.; Klenerman, D.Ultrasensitive Measurement of Ca(2+) Influx into Lipid Vesicles Induced by Protein Aggregates. *Angew. Chem. Int. Ed. Engl.* **2017**, *56* (27), 7750–7754.

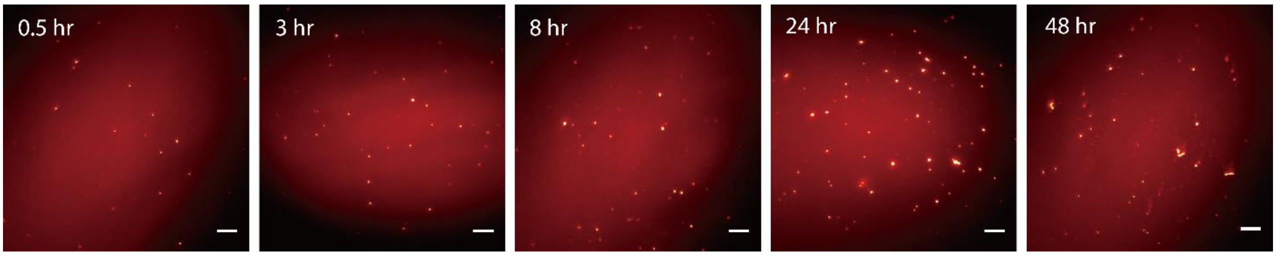

Supplementary Figure 1. Representative TIRF images of PrP aggregates at different times. The morphology of PrP aggregates at 0.5 hr, 3 hr, 8 hr, 24 hr, and 48 hr in unseeded aggregation was imaged using ThT. Monomeric PrP was incubated in a 1.5-mL microcentrifugal tube in the presence of 2 M GdnHCl at 37 °C with 200 rpm. At different time points, an aliquot was removed from the reaction mix, diluted to a final concentration of 300 nM, and loaded onto a PLL-coated glass coverslip in the presence of 25 µM ThT for TIRF imaging. The scale bars represent 10 µm.

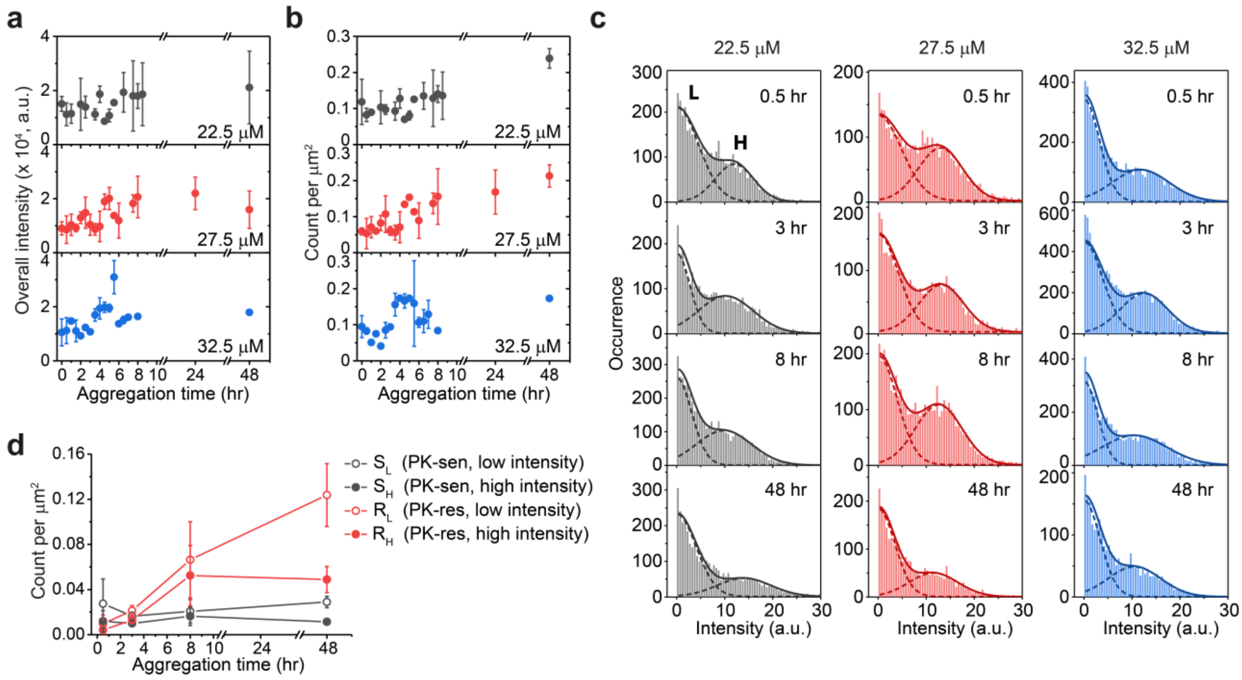

Supplementary Figure 2. Single-aggregate TIRF measurements of (a) overall ThT intensity and (b) number of PrP aggregates during fibril formation in the presence of 2 M GdnHCl. The measurements were carried out from three independent experiments by taking aliquots at various time points from the aggregation reaction mix that was incubated at 37 °C with shaking at 200 rpm. After dilution with 50 mM sodium phosphate (pH 7.0) in the presence of 25 µM ThT, the PrP aggregates were imaged on a TIRF microscope with a 3×3 image grid at 3 random positions (i.e. 27 simultaneous images). The images were analyzed with a Matlab-based script to identify individual aggregates. (c) Intensity histograms of ThT-active PrP aggregates at different time points. The PrP aggregates were split into two ThT intensity groups: low-intensity (L species) and high-intensity (H species). Illustrative fits of the individual PrP species (dash) to Gaussian functions and their accumulative fits (solid) are shown. The traces correspond to three independent experiments. (d) Temporal change in the number of the four identified ThT-active PrP species aggregated at 27.5 µM. Combining the intensity histograms and their PK resistance, the PrP aggregates were characterized into four species: low-intensity PK-sensitive (S_L), low-intensity PK-resistant (R_L), high-intensity PK-sensitive (S_H), high-intensity PK-resistant (R_H). The error bars represent standard deviations from three independent experiments.
